# Case Studies of the Synthesis of Bioactive Cyclodepsipeptide Natural Products

**DOI:** 10.3390/molecules18021337

**Published:** 2013-01-24

**Authors:** Sara C. Stolze, Markus Kaiser

**Affiliations:** Zentrum für Medizinische Biotechnologie, Fakultät für Biologie, Universitätsstraße 2, 45117 Essen, Germany

**Keywords:** cyclodepsipeptides, total synthesis, natural products, apratoxin A, spiruchostatin, FK228, ramoplanin, micropeptin T-20, Ahp-cyclodepsipeptide, symplocamide A

## Abstract

Cyclodepsipeptide natural products often display intriguing biological activities that along with their complex molecular scaffolds, makes them interesting targets for chemical synthesis. Although cyclodepsipeptides feature highly diverse chemical structures, their synthesis is often associated with similar synthetic challenges such as the establishment of a suitable macrocyclization methodology. This review therefore compiles case studies of synthetic approaches to different bioactive cyclodepsipeptide natural products, thereby illustrating obstacles of cyclodepsipeptide synthesis as well as their overcomings.

## 1. Introduction

Cyclodepsipeptide natural products are—as the name denotes—a class of natural products that contain a more or less pronounced peptidic part along with at least one ester bond in the macrocycle [1]. The complexity of cyclodepsipeptides can thereby vary from structurally relatively simple mono-macrocycles to highly complex architectures that often feature polyketide elements, non-proteinogenic amino acids or heterocyclic building blocks. Furthermore, intricate stereochemical settings can contribute to the overall complexity of a cyclodepsipeptide natural product.

Cyclodepsipeptides are usually biosynthesized *via* non-ribosomal assembly lines although recent research has revealed that some structurally complex peptidic (and thus also cyclodepsipeptidic) natural products are also generated by ribosomal peptide synthesis combined with post-translational modifications [2,3]. As cyclodepsipeptide natural products often show intriguing bioactivities, they have found wide application in chemical biology research as tools to study biomolecular interactions [4]; additionally, they have been explored extensively for biomedical applications [5,6]. In fact, several cyclodepsipeptide natural product families such as the didemnins, a class of structurally closely related cyclodepsipeptides with potent anticancer properties, have been intensively studied in the last years [7]. Further prominent examples are the histone deacetylase inhibitor largazole [8,9,10,11], the cytotoxic and potential anti-cancer agents of the cryptophicin family [12] or the anti-tumor cyclodepsipeptide kahalalide F [13,14,15].

As a prerequisite for further biological studies of a cyclodepsipeptide natural product, sufficient compound material is however required. Frequently, this demands for the development of efficient total synthesis methodologies as isolation procedures may only yield minute amounts of the natural product. Due to the complex structures of many cyclodepsipeptides, their synthesis is however often challenging. Nevertheless, some synthetic challenges are more or less common to many cyclodepsipeptide natural products. For example, all cyclodepsipeptide syntheses require the establishment of an appropriate ring closure methodology that in most cases will be achieved with either a macrolactonization or macrolactamization approach [16]. The cyclization reaction is however only one of the obstacles that are frequently encountered in the synthesis of structurally complex cyclodepsipeptide natural products. In this review, we therefore wish to present a number of case studies of total syntheses of bioactive cyclodepsipeptide natural products to illuminate common challenges associated with their synthesis and how they have been overcome.

## 2. Apratoxin A—The Challenge of Efficient Incorporation of Heterocylic Building Blocks

Apratoxin A is a 25-membered cyclodepsipeptide that was isolated alongside with two structurally related compounds, namely apratoxins B and C, from cyanobacterial *Lyngbya spp*. [17,18]. Apratoxin A thereby displayed a potent *in vitro* cytotoxic activity against human tumor cell lines with a so far unknown mode-of-action [18]. Structurally, apratoxin A features peptide and polyketide components: The polyketide part of this natural product is represented by the hydroxylated fatty acid moiety 3,7-dihydroxy-2,5,8,8-tetramethylnonanoic acid (*Dtena*) that forms an ester bond with a proline residue. Besides proline, it furthermore contains two *N*-methylated amino acids and an α,β-unsaturated fragment with a thiazoline moiety that originates from cysteine. In fact, heterocyclic components that are biosynthetically derived from amino acids are frequently found structural elements in many cyclodepsipeptide natural products. Naturally, an integral part of a total synthesis of apratoxin A is therefore the development of an efficient approach to incorporate the heterocyclic moiety into the cyclodepsipeptide ring structure.

The first total synthesis of apratoxin A was achieved in 2003 by Forsyth and coworkers using an intramolecular Staudinger/aza-Wittig reaction at a late stage of the synthesis to establish this thiazoline moiety from an azide-modified cysteine derived precursor [19]. Indeed, the development of a suitable methodology for the generation of the thiazoline residue turned out as one of the bottlenecks of their apratoxin A synthesis [19,20,21].

**Scheme 1:**
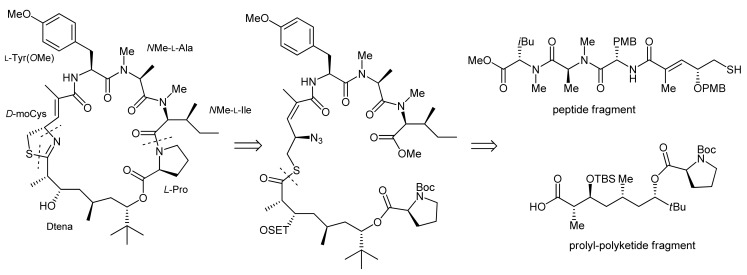
Forsyth’s retrosynthesis of apratoxin A [19,20,21].

To achieve the total synthesis of this natural product, Forsyth *et al.* made three major retrosynthetic disconnections (Scheme 1): the ultimate ring closure by a macrolactamization between the proline and *N*-methyl isoleucine residues, the formation of the thiazoline moiety from an azide and a thioester residue and, finally, the fragmentation of the linear precursor molecule at the thioester linkage into a peptide portion and a polyketide-prolyl ester building block.

**Scheme 2:**
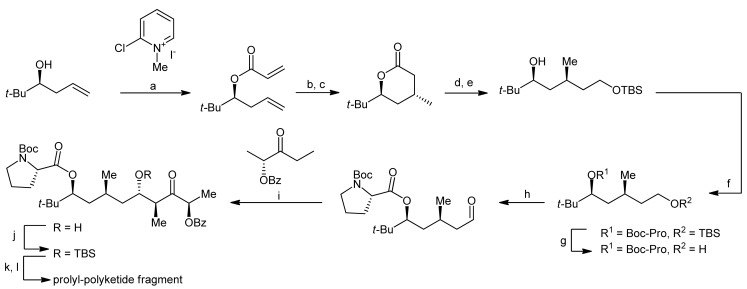
Synthesis of the prolyl-polyketide fragment of apratoxin A [19,20,21].

The synthesis of the prolyl-polyketide fragment started with the acylation of a homoallylic alcohol with acrylic acid thereby yielding a diene that could be converted to an α,β-unsaturated δ-valeryl lactone by a ring closing metathesis reaction (Scheme 2).

In the next step, a cuprate-mediated addition was used to install a methyl group in a regio- and stereoselective manner in the β-position. After a reductive ring-opening of the lactone and the subsequent protection of the resulting primary alcohol as a silyl ether, the proline moiety was introduced by esterification of the secondary alcohol using Yamaguchi conditions. In order to establish the complete polyketide framework, the primary alcohol was then converted to an aldehyde in a deprotection-oxidation protocol and the resulting aldehyde was subjected to an anti-selective aldol reaction with (*R*)-benzyloxy-3-pentanone, thereby yielding the β-hydroxy ketone. The newly established secondary alcohol was subsequently protected as a TBDMS ether, and the chiral directing functionality was cleaved in an oxidative manner to afford the desired carboxylic acid.

**Scheme 3:**
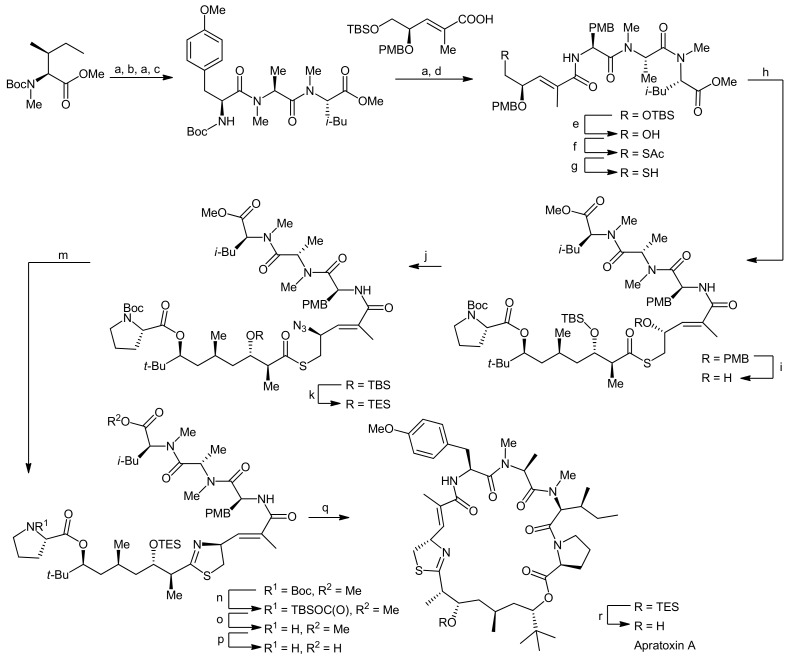
Final assembly of apratoxin A by Forsyth *et al.* [19,20,21].

The synthesis of the peptide portion started from Boc-NMe-Ile-OMe, which was subjected to a sequential deprotection-coupling protocol, installing first *N*-methylalanine, followed by a methylated tyrosine residue and finally a cysteine surrogate to afford the tetrapeptide fragment (Scheme 3). To ensure a good coupling efficiency, especially to the sterically hindered amino acids, PyAOP was used as the coupling reagent. To establish the desired terminal thiol functionality, the terminal silyl ether was first deprotected and the resulting hydroxyl function was replaced by a thioacetate moiety in a Mitsunobu reaction. The thiol group was then liberated by basic hydrolysis of the acetate and could subsequently be coupled to the prolyl-polyketide fragment using diphenylphosphoryl azide (DPPA) as the activator to afford a thiol ester. To set the stage for the desired aza-Wittig reaction, the γ-PMB ether was next converted to an alcohol which was subsequently replaced by an azide using Mitsunobu conditions. Prior to the thiazoline formation an exchange of the TBDMS ether to a TES ether was required because the TBDMS ether could not be cleaved at a late stage of the synthesis in a previous attempt to synthesize apratoxin A. This adjustment then set the stage for the Staudinger/aza-Wittig reaction which afforded the linear thiazoline-containing macrocyclization precursor. Finally, the termini of the linear precursor were deprotected and the macrocyclization reaction was carried out using PyAOP as the coupling reagent. The removal of the remaining TES ether then yielded apratoxin A.

**Scheme 4:**
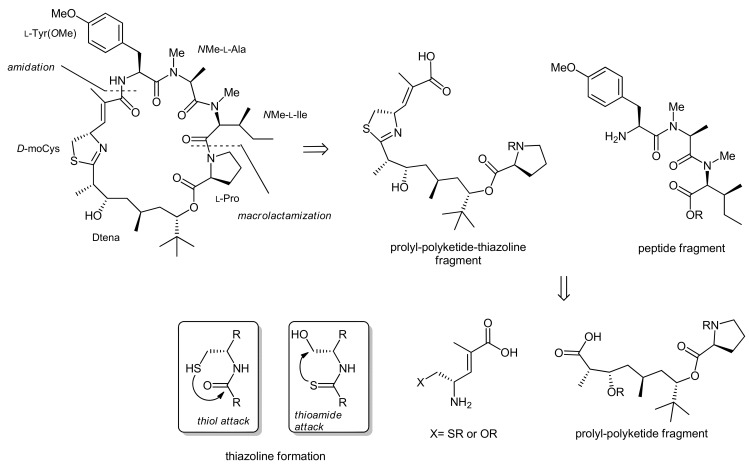
Doi’s retrosynthesis of apratoxin A [22,23].

**Scheme 5:**
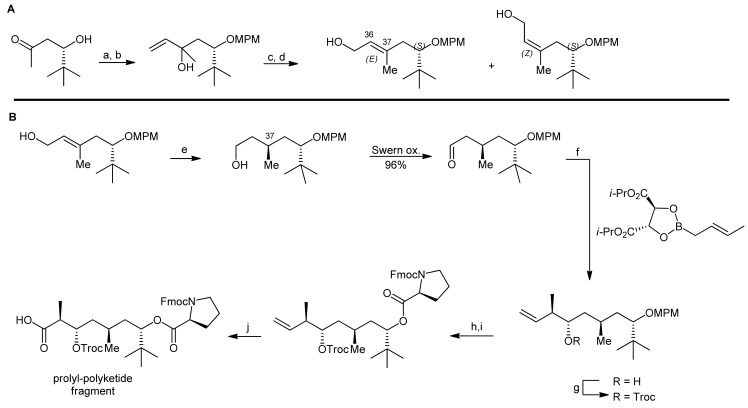
Asymmetric synthesis of the prolyl-polyketide fragment by Doi *et al*. [23].

An alternative synthetic strategy to apratoxin A was presented by Doi and coworkers that aimed to develop a more flexible approach to this natural product as well as to related analogs [22,23]. Their synthetic strategy therefore involved an earlier introduction of the thiazoline residue (Scheme 4). They therefore decided to first synthesize a prolyl-thiazoline-polyketide fragment which was then coupled to a tripeptide building block, followed by a macrolactamization (that was carried out between proline and N-methylisoleucine as in the Forsyth approach). For installation of the thiazoline moiety, two different approaches were investigated: In the first approach, the thiazoline moiety was formed using Kelly’s methodology, *i.e.*, a cysteine side chain thiol attacking the adjacent amide carbonyl group followed by dehydration [22]. Alternatively, the synthesis of a thiazoline *via* a thioamide intermediate was explored (Scheme 4, boxes). However, this approach failed as the required thioamide could not be generated. Nevertheless, this ‘failed’ route was instead used to synthesize an oxazoline analog of apratoxin A [23]. Along these lines, their apratoxin A synthesis started with the generation of the polyketide portion and the four stereocenters of this fragment were constructed by a sequence of three asymmetric reactions (Scheme 5). The starting material was prepared by a proline-catalyzed aldol reaction from pivaldehyde and acetone (Scheme 5, **A**). After the protection of the terminal hydroxyl function, a chain elongation *via* a Grignard reaction with vinyl magnesium bromide was carried out. The resulting secondary allylic alcohol was then isomerized with a rhenium catalyst, leading to a mixture of (*E*)- and (*Z*)-isomeric primary allylic alcohols in 42% and 44% yield.

**Scheme 6:**
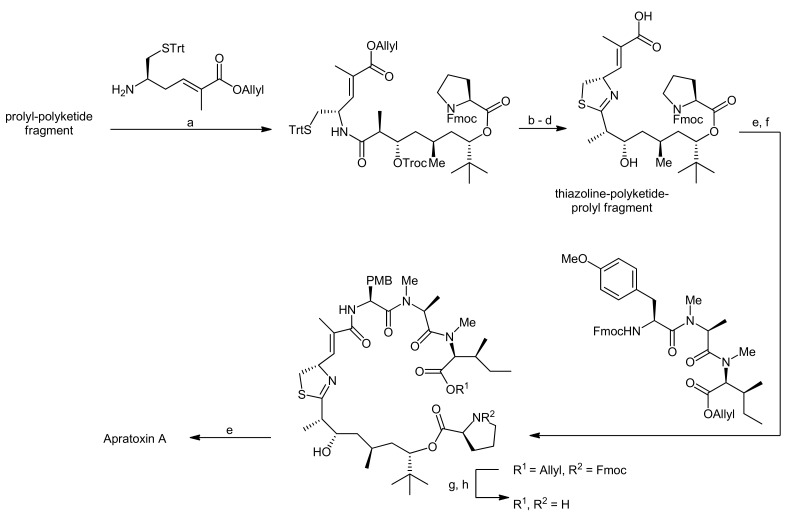
Heterocycle formation and final assembly to apratoxin A [23].

The isomers were separated by column chromatography and were individually subjected to asymmetric hydrogenation to establish the chiral center at C37 (Scheme 5, **B**). For obtaining the desired (*R*)-configuration at C37, the (*E*)-isomer was hydrogenated in presence of 2 mol% Ru(OAc)_2_[(*S*)-binap]; for the (*Z*)-isomer, Ru(OAc)_2_[(*R*)-binap] was used. After conversion of the primary alcohol to an aldehyde by Swern oxidation, the two final stereocenters were installed by a crotylborylation reaction, using an (*E*)-crotylborane reagent. After protection of the secondary alcohol with a Troc group, the MPM group was removed under oxidative conditions and the resulting alcohol was esterified with Fmoc-Pro-OH using the Yamaguchi protocol. Finally, the terminal alkene was converted to a carboxylic acid by a two-step oxidation protocol using first oxone/OsO_4_ and then NaIO_4_ in a one-pot reaction.This prolyl-polyketide fragment was then coupled with the cysteine-derived α,β-unsaturated *moCys* building block synthesized by the Wittig methodology (Scheme 6).

Then, the thiazoline was generated by Kelly’s dehydrative methodology. To this end, the precursor was treated with Ph_3_PO/Tf_2_O, yielding the desired thiazoline after Troc removal in 90% yield over two steps. Removal of the allyl group then provided the thiazoline-polyketide-prolyl fragment that was subsequently coupled with a H-Tyr(OMe)-NMe-Ala-NMe-Ile-OAllyl tripeptide with HATU as a coupling agent. After removal of the allyl ester and the Fmoc group, the macrolactamization was carried out using high dilution macrocyclization conditions to afford apratoxin A in 72% yield.

**Figure 1:**
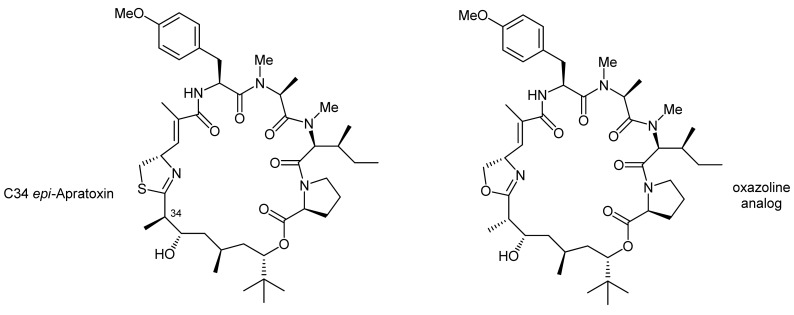
Apratoxin derivatives prepared by the Doi group [22,23].

The C34 *epi*-apratoxin A was synthesized in an analogous fashion using an individually prepared C34 *epi*-prolyl-polyketide fragment (Figure 1). In addition, an oxazoline variant could also be generated, using a *moSer* building block instead of a *moCys* building block (Figure 1). The formation of an oxazoline instead of a thiazoline moiety was thereby achieved with the reagent DAST [23].

For completeness, it should be noted that also the groups of Ma and Liu have reported a synthesis of apratoxin A and analogs, while the Luesch group has published the preparation of some apratoxin analogs [24,25]. Finally, a total synthesis of apratoxin D that was isolated in 2008 and shows strong structural similarities to apratoxin A was recently reported [26,27].

## 3. Spiruchostatin A and FK288: The Challenges to Find Efficient Macrocylization Routes

Spiruchostatin A and B, FK228 and also the natural product largazole are cyclodepsipeptide natural products that potently inhibit histone deacetylases (HDACs). Inhibition is thereby achieved by a coordination of their (*S*)-3-hydroxy-7-mercaptohept-4-enoic acid moiety to the active site Zn^2+^ residue of HDACs [9,10]. In FK228 and the spiruchostatins, an *in vivo* reduction of the disulfide bond generates the required ‘free’ thiol moiety, whereas the formation of “largazole thiol” is achieved by *in vivo* hydrolysis of largazole’s octanoic acid thioester [10]. Due to their interesting biological properties, the synthesis of these cyclodepsipeptides has been investigated heavily; these studies have revealed that the choice of a proper macrocyclization methodology is highly important for an efficient synthesis. As synthetic approaches to largazole have recently been reviewed [9,16], we will however focus in our overview on spiruchostatin A and FK228 synthesis.

Spiruchostatin A was isolated from *Pseudomonas* sp. and its bicyclic depsipeptide core comprises (3*S*, 4*R*)-statine, d-cysteine, d-alanine and the prominent (*S*)-3-hydroxy-7-mercaptohept-4-enoic acid as building blocks [28]. The bicyclic system is thereby created by an ester linkage between the statine and the mercaptoheptenoic acid residues; the latter one is also involved in the formation of the second linkage by forming a disulfide bridge to the cysteine residue.

**Scheme 7:**
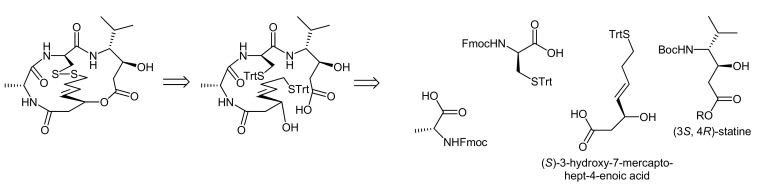
Retrosynthetic disconnections of spiruchostatin A [29].

To demonstrate the challenge of finding an efficient macrocylization route, we will concentrate our discussion on the spiruchostatin A synthesis by Takahashi and coworkers that disconnected spiruchostatin A into 4 different building blocks (a d-cysteine, a d-alanine and a statine building block and (*S*)-3-hydroxy-7-mercaptohept-4-enoic acid) (Scheme 7) [29].

**Scheme 8:**
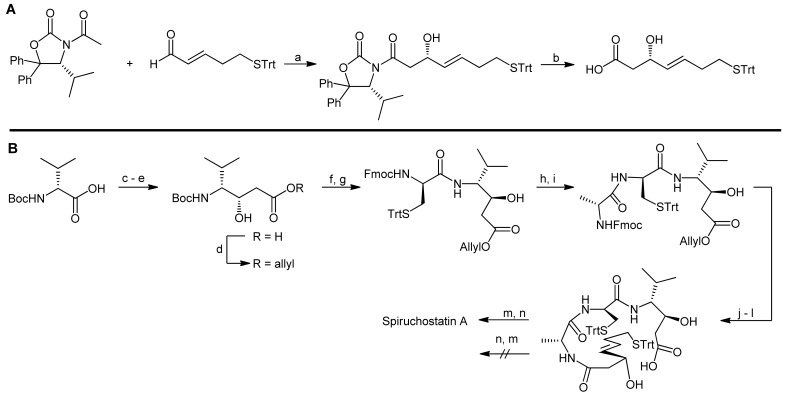
Synthesis of a mercaptoheptenoic acid building block (**A**) and assembly of spiruchostatin A (**B**) [29].

Accordingly, a reliable access to (*S*)-3-hydroxy-7-mercaptohept-4-enoic acid was first required and the authors proposed to obtain this β-hydroxy acid from the corresponding aldehyde in an acetate aldol reaction (Scheme 8, **A**). As they furthermore assumed that an acetate aldol reaction with the classical Evans’ oxazolidin-2-one auxiliary would proceed with only poor selectivity, they used Seebach´s *N*-acetyl-oxazolidin-2-one auxiliary [29].

The (3*S*,4*R*) statine building block was obtained from D-valine using a malonate ester methodology, followed by a stereoselective reduction (Scheme 8, **B**). The overall synthesis strategy required the introduction of a protecting group compatible with both Fmoc- and Boc-removal and, therefore, an allyl ester was chosen. The statine building block was then deprotected and coupled with trityl-protected cysteine. In the following two steps, D-alanine and the protected (*S*)-3-hydroxy-7-mercaptohept-4-enoic acid were coupled, resulting in the generation of the acyclic precursor molecule. Finally, different strategies for cyclization to the bicyclic structure were investigated, *i.e.*, a formation of the macrocycle a) prior to the disulfide bridging or b) after the disulfide bridging. Intriguingly, a macrolactonization of the disulfide bridged molecule could not be achieved under the conditions explored. In contrast, macrolactonization followed by disulfide formation delivered the desired bicycle (Scheme 8, **B**) [29]. Macrolactonization of the acyclic precursor was thereby achieved by the Shiina method, in which 2-methyl-6-nitrobenzoic anhydride is used at room temperature and that has proven to be a valuable method for macrocyclization reactions, often resulting in better yields and higher monomer contents than ‘classical’ Yamaguchi conditions [30]. In the spiruchostatin A synthesis, Shiina´s conditions afforded the desired product in 67% yield after disulfide formation, whereas Yamaguchi´s conditions resulted in yields of only 40%. Interestingly, the same group has recently also reported a semi-solid phase synthesis of spiruchostatin A [31] in which the critical ring closures were however still conducted in solution phase. In addition, a total synthesis of spiruchostatin B *via* an automated synthesizer has also been performed by this group, again relying on the established methodology [32]. For completeness, it should be noted that various additional syntheses of spiruchostatin A and B have been reported so far [29,33,34,35,36].

**Figure 2:**
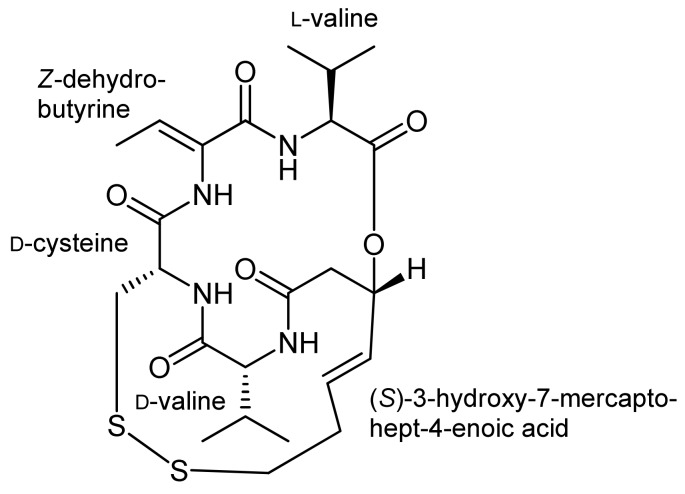
Chemical structure of the histone deacetylase inhibitor FK228.

Different ring closing strategies were also pondered for synthetic approaches towards the histone deacetylase inhibitor FK228 (also known as FR-901,228) that was isolated from *Chromobacterium violaceum* [37,38,39]. Besides the (*S*)-3-hydroxy-7-mercaptohept-4-enoic acid moiety, FK228 also contains a cysteine residue that forms a disulfide with the thiol of the mercaptoheptenoic acid, a (*Z*)-dehydrobutyrine moiety as well as D-valine and L-valine residues (Figure 2). The latter one forms an ester bond with the hydroxyl functionality of the mercaptoheptenoic acid to establish the bicyclic framework of FK228. Several syntheses of this important cyclodepsipeptide have so far been developed [35,40,41,42,43,44]. As for spiruchostatin, in these syntheses, the ring closure to the bicyclic ring system turned out to be the major challenge.

**Scheme 9:**
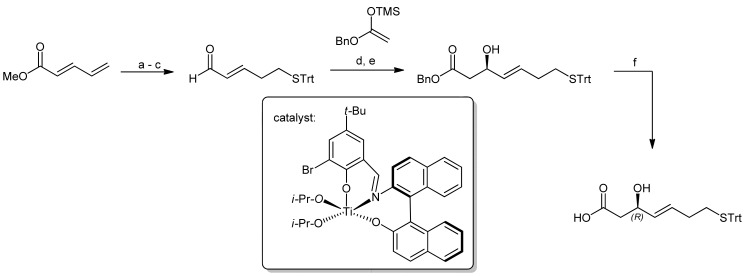
Synthesis of the mercaptoheptenoic acid building block using Carreira’s asymmetric aldol addition [40].

The first synthesis of this natural product was reported by Simon *et al.*, in which the required mercaptohepenoic acid residue was synthesized using Carreira’s asymmetric aldol addition with a chiral Ti(IV) ligand derived from binaphtyl amino alcohol (Scheme 9) [40]. The reaction delivered the desired (*R*)-configured secondary alcohol (because ring closure was achieved by a Mitsunobu reaction) in an excellent yield and with high enantioselectivity.

**Scheme 10:**
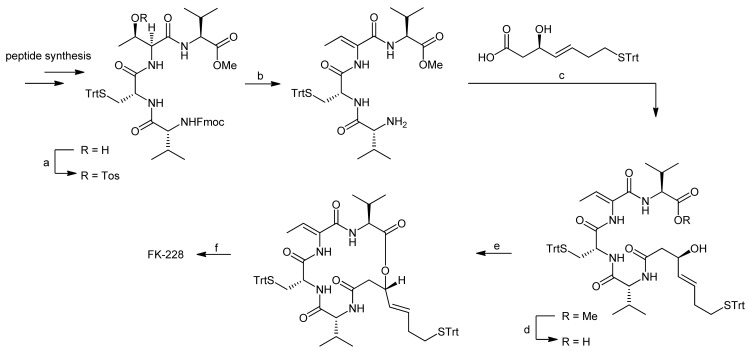
Final steps of the synthesis of FK228 by Simon *et al*. [40].

The peptide portion of FK228 was subsequently synthesized using standard Fmoc peptide chemistry (Scheme 10) [40].

After the assembly of the sequence Fmoc-D-Val-D-Cys(Trt)-L-Thr-L-Val-OMe, the secondary alcohol of the threonine side chain was activated by tosylation and underwent elimination to the (*Z)*-dehydrobutyrine moiety upon treatment with DABCO; an addition of diethylamine affected simultaneous Fmoc removal. Subsequently, the mercaptoheptenoic acid building block was attached. Basic hydrolysis of the methyl ester of the C-terminal valine then set the stage for a ring closure by macrolactonization by a Mitsunobu approach. The conditions for this step were optimized extensively and finally yielded the FK228 precursor in 62% yield. An addition of TsOH was thereby found crucial to suppress elimination of the activated allylic alcohol. Oxidative intramolecular coupling of the sulfur groups using I_2_/MeOH oxidation finally yielded FK228, with an overall yield of 18% after 14 steps.

After the assembly of the sequence Fmoc-D-Val-D-Cys(Trt)-L-Thr-L-Val-OMe, the secondary alcohol of the threonine side chain was activated by tosylation and underwent elimination to the (*Z)*-dehydrobutyrine moiety upon treatment with DABCO; an addition of diethylamine affected simultaneous Fmoc removal. Subsequently, the mercaptoheptenoic acid building block was attached. Basic hydrolysis of the methyl ester of the C-terminal valine then set the stage for a ring closure by macrolactonization by a Mitsunobu approach. The conditions for this step were optimized extensively and finally yielded the FK228 precursor in 62% yield. An addition of TsOH was thereby found crucial to suppress elimination of the activated allylic alcohol. Oxidative intramolecular coupling of the sulfur groups using I_2_/MeOH oxidation finally yielded FK228, with an overall yield of 18% after 14 steps.

**Scheme 11:**
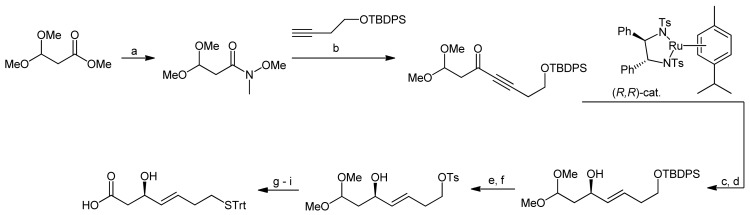
Synthesis of the mercaptoheptenoic acid building block by Williams *et al.* [41].

More than a decade after the first synthesis of FK228, Williams and coworkers set out to improve the original synthesis by Simon *et al.* because they had encountered serious problems when trying to reproduce the reactions of the original synthesis approach [41]. To this end, they first investigated an alternative approach to (*S*)-3-hydroxy-7-mercaptohept-4-enoic acid-containing natural products. While most syntheses of this building block were based on efficient asymmetric aldol approaches [29,33,45], Williams and coworkers used an asymmetric hydrogen transfer reaction (Scheme 11) [41]. In the first step of their synthesis approach, commercially available methyl 3,3-dimethoxypropionate was converted to the Weinreb amide under standard conditions. Addition of a lithium acetylide to the amide then yielded a propargyl ketone. Using Noyori’s asymmetric transfer hydrogenation, the ketone was subsequently reduced to afford the corresponding (*R*)-propargylic alcohol, followed by treatment with Red-Al, yielding exclusively the (*E*)-alkene. The subsequent establishment of the acid function and conversion of the terminal silyl ether to the trityl mercaptane required delicate handling to avoid oxidation of the mercaptane. Accordingly, the silyl ether was first deprotected and converted to a tosylate. After this step, the dimethyl acetal was hydrolyzed to the aldehyde and immediately converted to the acid using Pinnick oxidation conditions. In the final step, the trityl mercaptane moiety was then introduced *via* a nucleophilic displacement of the tosylate residue, yielding the desired mercaptoheptenoic acid in an overall yield of 13.3%.

Having successfully established a synthesis route for the mercaptoheptenoic acid, Williams *et al.* then set out to improve the assembly of FK228 in terms of protecting groups and the yields of the individual reactions [41]. Although they achieved slight improvements for some steps, the overall synthetic strategy including ring closure by a macrolactonization resembled the original approach in most steps. Despite intense efforts, they therefore were not able to significantly improve the yield-limiting ring closure reactions.

**Scheme 12:**
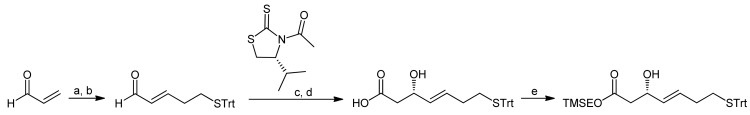
Synthesis of the mercaptoheptenoic acid building block by the Ganesan group [43].

Due to the difficulties to establish efficient macrolactonization conditions, Ganesan and coworkers (who had previously worked also on the synthesis of Spiruchostatins [29,33]) subsequently explored a macrolactamization approach [43]. For the synthesis of the mercaptoheptenoic acid, they adapted the Wentworth-Janda protocol that had been developed during the synthesis of the natural product FR901,375 [45]. The new protocol provided the mercaptoheptenoic acid in 4 steps from acrolein [43], using the Fujita-Nagao thiazolidinedione auxiliary that is selective in acetate aldol reactions (Scheme 12) [46,47]. In order to close the ring by a macrolactamization reaction, the mercaptoheptenoic acid needed to be protected orthogonally; consequently, after the hydrolysis of the aldol auxiliary the resulting free acid was protected as a 2-(trimethylsilyl)ethyl (TMSE) ester.

The synthesis of the linear depsipeptide was initially planned as a straightforward sequential approach starting from L-valine and thus involved the introduction of the ester bond between L-valine and the mercaptoheptenoic acid as the ultimate step. The synthesis of a precursor tetrapeptide as well as the activation and subsequent elimination of the threonine side chain to afford the dehydrobutyrine proceeded smoothly. The esterification reaction however turned out to be as demanding as the previously examined macrolactonization reactions. Investigation of several reaction conditions showed that 1-(2-mesitylensulfonyl)-3-nitro-1*H*-1,2,4-triazole (MSNT) afforded the depsipeptide in 34% yield and recovered 42% of unreacted alcohol starting material. Elongation of reaction times however did not improve yields and resulted in epimerization. Sterical hindrance was identified as the most likely reason for this inefficient coupling.

**Scheme 13:**
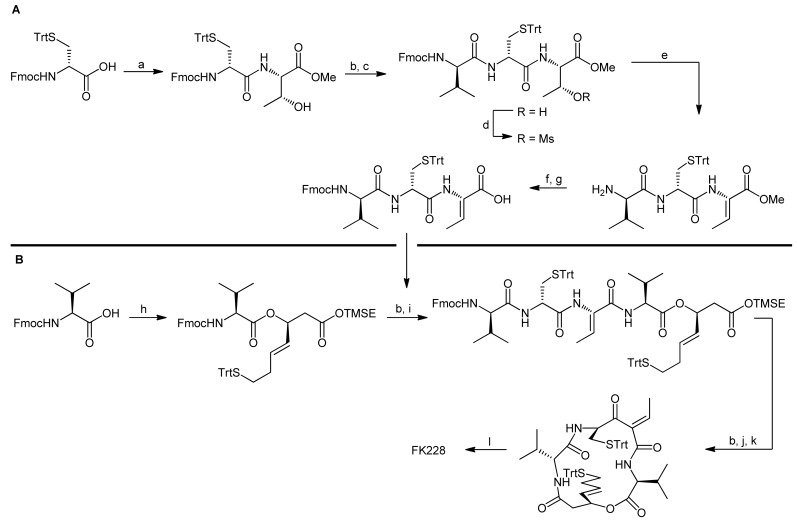
Assembly of FK228 by the Ganesan group following a macrolactamization approach [43].

Consequently, the synthesis of the linear depsipeptide was reconsidered and the ester bond between L-valine and mercaptoheptenoic acid was instead installed in the first step of the synthesis (Scheme 13, **B**) [43]. The carbodiimide-mediated esterification reaction then afforded the depsipeptide in 85% yield. The remaining tripeptide fragment was synthesized starting from Fmoc-D-Cys(Trt)-OH, which was first coupled C-terminally with Fmoc-L-Thr-OMe and then *N*-terminally with Fmoc-D-Val-OH (Scheme 13, **A**). Mesylation and DABCO-mediated dehydration of the threonine side chain then afforded the tripeptide incorporating the dehydrobutyrine moiety. After hydrolysis of the methyl ester and reinstallation of the Fmoc protecting group that was cleaved during the elimination reaction, the acid tripeptide fragment was coupled to the N-terminally deprotected depsipeptide. The resulting acyclic precursor was then fully deprotected and cyclized successfully using HATU as coupling reagent. The final oxidative disulfide formation proceeded smoothly to yield the desired natural product FK228 in overall high yields. In fact, the scope of the synthesis was elegantly demonstrated by a gram-scale FK228 production [43]. 

## 4. Ramoplanin Aglycons: The Challenges to Properly Assemble Cyclodepsipeptides from Smaller Fragments

**Figure 3:**
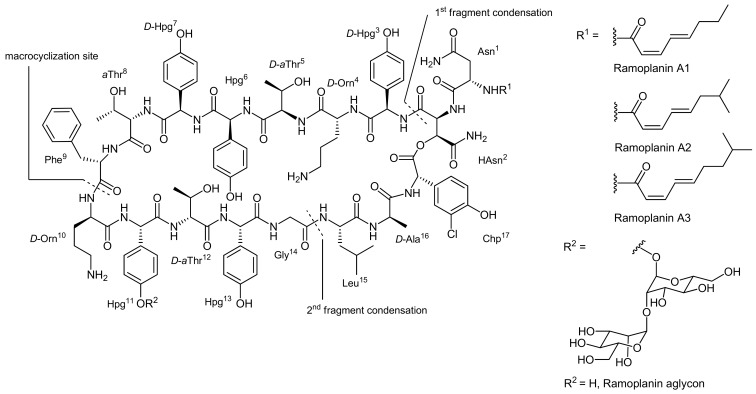
Fragmentation of Ramoplanin for a convergent synthesis approach [50].

Ramoplanins are closely related, rather large lipoglycodepsipeptides isolated from the fermentation broth of *Actinoplanes* sp. ATCC33076 that differ by diverse lipid chains attached to their Asn *N*-terminus by acylation [48]. They are built up from a 49-membered depsipeptide core that consists of 17 amino acids (Figure 3); 13 of the amino acids are non-proteinogenic with many of them being racemization prone phenylglycine derivatives. Moreover, seven amino acids display a D-configuration. The ester bond that constitutes the cyclodepsipeptide is formed between the C-terminal 3-chloro-4-hydroxyphenylglycine (Chp^17^) residue and the hydroxy group of a β-hydroxyasparagine (β-OH-Asn^2^) moiety.

The glycosidic part of the molecule consists of two mannose units linked to the phenol residue of one hydroxyphenylglycine unit (Hpg^11^). The ramoplanins are also related to ramoplanose which bears the same lipid side chain as ramoplanin A2 and possesses a glycosidic part consisting of three mannose subunits [49]. The ramoplanin complex (*i.e.*, the natural mixture of ramoplanins A1 to A3) displays very potent activity against a wide range (500 strains) of Gram-positive bacteria, including methicillin-resistant enterococci and all known strains of methicillin-resistant *Staphylococcus aureus* (MRSA). Indeed, the complex has been found to be 2 to 10 times more active than the ‘last resort’ antibiotic vancomycin for the tested strains [50]. Similar to other antibiotic compounds, the ramoplanins interfere with the bacterial cell wall synthesis and their molecular mode-of-action has recently been reviewed [48]. Importantly, ramoplanins also display an activity against lately emerged vancomycin-resistant strains and are currently in clinical trials for a number of indications. The aglycons of all ramoplanins displayed a similar or even stronger biological activity; consequently, efforts towards the synthesis of the aglycons were driven forward. The lipid part of the ramoplanins is attached to the amine of Asn^1^ by acylation.

The first total synthesis of a ramoplanin aglycon, namely the ramoplanin A2 aglycon, was reported by Boger and coworkers that due to the exceptional size of this natural product sought a highly convergent synthesis [50,51]. The molecule was therefore divided into three key subunits that were synthesized individually and fused later to form the macrocycle (Figure 3). The chosen peptidic fragments were a heptapeptide (residues 3–9), a pentadepsipeptide (residues 1, 2 and 15–17) and a pentapeptide (residues 10–14); in order to prevent racemization during assembly of the fragments, no disconnections were thereby made at the C-terminal end of phenylglycines. 

**Scheme 14:**
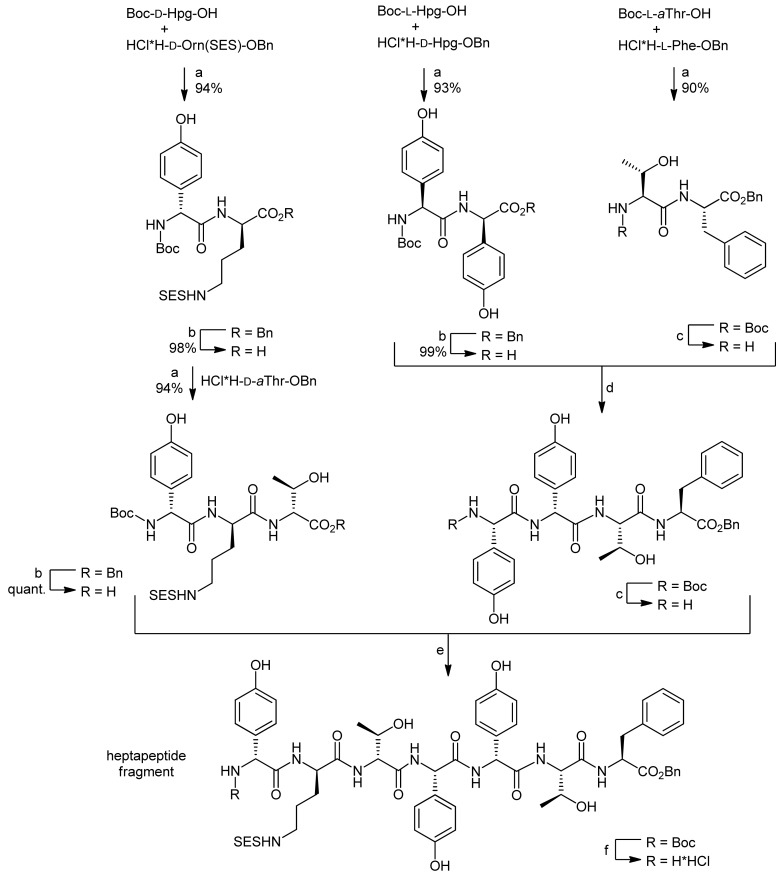
Construction of the heptapeptide fragment by Boger *et al.* [50,51].

The heptapeptide fragment was synthesized by the fusion of an N-terminal tripeptide and a C-terminal tetrapeptide (Scheme 14) [50,51]. The tripeptide synthesis started from Boc-D-Hpg-OH which was coupled with the hydrochloride of D-Orn(SES) benzyl ester (SES = 2-(trimethylsilyl)ethylsulfonyl) using EDC/HOAt activation. After deprotection of the C-terminus by benzyl ester hydrogenolysis, a D-*a*Thr benzyl ester hydrochloride was coupled to the dipeptide using EDC/HOAt activation to afford the protected tripeptide in 94% yield.

The tetrapeptide was synthesized by coupling of the two dipeptides Boc-L*-*Hpg-D-Hpg-OH and H-L-*a*Thr-L-Phe-OBn using DEPBT/NaHCO_3_ activation to prevent racemization of the sensitive phenylglycine; the tetrapeptide was obtained in 83% yield. Neither racemization of the sensitive Hpg units nor β-elimination of the hindered *a*Thr sidechain was thereby observed. After Boc deprotection of the N-terminus, the tetrapeptide was then coupled to the C-terminally deprotected tripeptide using EDC/HOAt activation to yield the heptapeptide that was subsequently deprotected at the N-terminus for further coupling. 

**Scheme 15:**
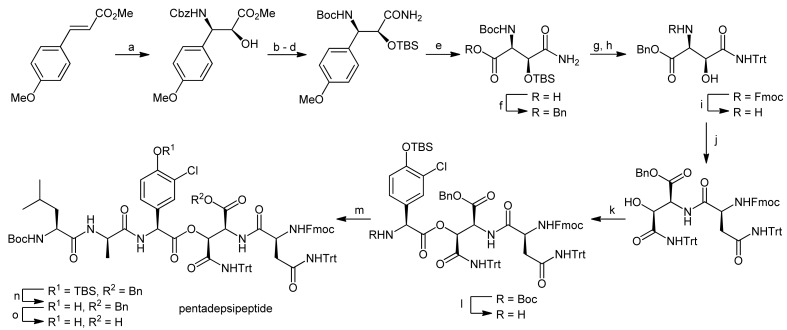
Synthesis of the pentadepsipeptide fragment [50,51].

The key element of the pentadepsipeptide synthesis was the construction of an orthogonally protected L-threo-β-hydroxyasparagine residue and its subsequent esterification with Chp17 (Scheme 15). For the synthesis of the required L-threo-β-hydroxyasparagine moiety, a novel methodology was developed [52]. To this end, a styrene-substituted anisole was subjected to a Sharpless asymmetric aminohydroxylation (Scheme 15) [50,51]. By careful adjustment of the amount of (DHQD)_2_PHAL used in this reaction, an *e.e.* of 99% could be achieved. The hydroxyl function was then protected as a silyl ether and the Cbz-protection of the amino group was replaced by a Boc group. Finally, the methyl ester was converted into an amide. Oxidative cleavage of the anisole using 5% RuCl_3_ and NaIO_4_ afforded the free acid derivative which was then protected as a benzyl ester. For the desired building block, the Boc and TBS groups were removed under acidic conditions and the amine was re-protected with an Fmoc group; the side chain amide was protected with a trityl group. After removal of the Fmoc protecting group, the building block was then coupled with Fmoc-L-Asn(Trt)-OH using EDC/HOAt activation to yield the dipeptide in 90% yield. To optimize the esterification of this dipeptide with Boc-L-Chp(TBS)-OH, different conditions were explored, but only moderate conversion rates and low diastereoselectivities were achieved. Finally, EDC activation with DMAP as a catalyst at low temperatures delivered the ester in 87% yield with a diastereoselection of >10:1 for the desired isomer. The Boc group was cleaved and the resulting tridepsipeptide was coupled to the Boc-L-Leu-D-Ala-OH dipeptide. Finally, the TBS protecting group and the benzyl ester on the hydroxyasparagine acid function were removed, thereby yielding the first fragment for the assembly of ramoplanins.

**Scheme 16:**
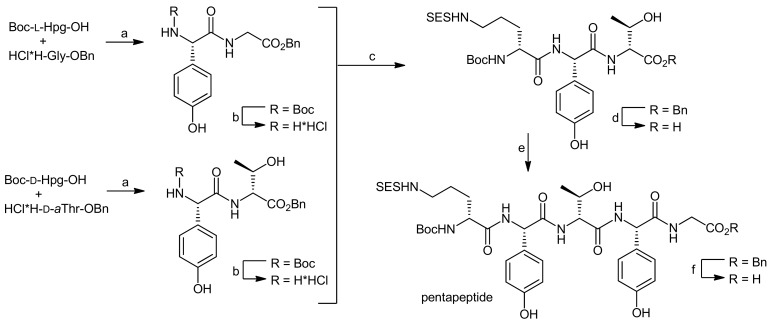
Convergent synthesis of the pentapeptide fragment of ramoplanin [50,51].

The (10–14) pentapeptide was subsequently synthesized in a convergent manner by fragment fusion (Scheme 16) [50,51]. Boc-L-Hpg-OH was coupled with H-D-*a*Thr-OBn hydrochloride using DEPBT activation, again without any detectable racemization. After removal of the Boc protecting group, the resulting hydrochloride was coupled to Boc-D-Orn(SES)-OH. After hydrogenolysis of the C-terminal benzyl ester, this tripeptide was coupled with H-L-Hpg-Gly-OBn (obtained by DEPBT-mediated coupling of Boc-L-Hpg-OH with glycine benzyl ester hydrochloride and subsequent Boc deprotection). The resulting pentapeptide was finally deprotected at the C-terminus for later coupling reactions.

**Scheme 17:**
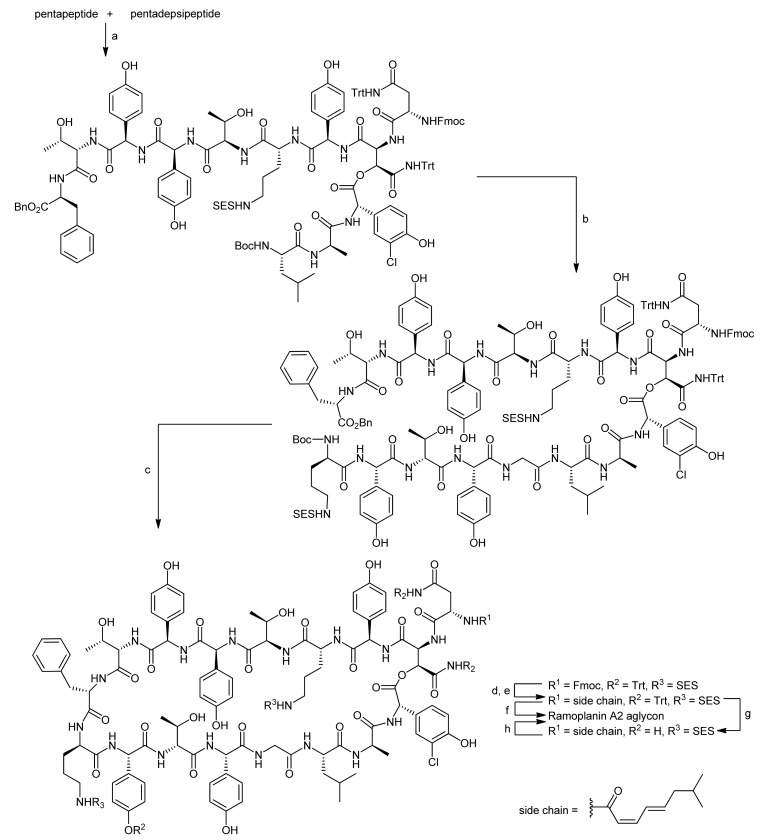
Assembly of the ramoplanin A2 aglycon [50,51].

To assemble the depsipeptide core structure, the free N-terminus of the pentapeptide and the pentadepsipeptide deprotected at the hydroxyasparagine’s acid function were coupled using DEPBT activation to afford the corresponding decapeptide in yields between 50%–68%, importantly without any competitive β-elimination products (Scheme 17) [50,51]. After deprotection of the N-terminus of the decapeptide with BCB (*B*-bromocatecholborane), the heptapeptide was coupled to obtain the linear macrocyclization precursor in yields from 60%–82%. After Boc deprotection and benzyl ester hydrogenolysis, the macrocyclization reaction was finally carried out using EDC/HOAt activation, providing the macrocyclic depsipeptide in yields of 54%–72% (3 steps). Consequently, only the acyl side chain introduction remained to finish the synthesis of the ramoplanin A2 aglycon. However, the removal of the Fmoc, SES and trityl groups in the presence of the sensitive depsipeptide ester turned out to be more challenging than expected. Tedious model examinations finally provided a suitable strategy for completing the total synthesis.

First, the Fmoc group was removed with TBAF using specially developed conditions; then the acyl side chain was introduced into the target molecule. The fully deprotected ramoplanin A2 aglycon was then obtained either by a one-step simultaneous HF cleavage of the SES and Trt groups or by a sequential Trt cleavage mediated by TFA followed by HF cleavage of the SES groups. Both strategies provided the ramoplanin A2 aglycon, identical in all respects with an original sample, in satisfying yields of 82% and 83%, respectively. In a follow-up synthesis, Boger and coworkers were also able to synthesize the aglycons of the minor components of the ramoplanin complex, *i.e.*, the ramoplanin A1 aglycon and the ramoplanin A3 aglycon, again employing their highly convergent strategy [53].

## 5. Syntheses of 3-Amino-6-hydroxy-2-piperidone (Ahp) Cyclodepsipeptides: The Challenge of Synthesizing Cyclodepsipeptides with Chemically Instable Elements

The class of Ahp cyclodepsipeptides has entered the focus of researchers due to the intriguing mode-of-action displayed by its members: the Ahp cyclodepsipeptides have been shown to specifically and potently inhibit S1 (trypsin- and chymotrypsin-like) serine proteases in a non-covalent manner [54,55,56]. As a consequence, the Ahp cyclodepsipeptides have been suggested as possible leads for the development of novel protease inhibitors [57].

All members of this natural product class share a 19-membered cyclodepsipeptide core that incorporates besides the eponymous 3-amino-6-hydroxy-piepridone (Ahp) unit, an N-methylated aromatic amino acid as well as the depsipeptide linkage between the hydroxyl group of a threonine and an aliphatic amino acid (Val or Ile). The threonine amine serves as an attachment point for different side chains; here, a high level of variation can be observed [58,59,60,61,62]. The Ahp moiety which due to its hemiaminal structure is a potentially chemically instable functional group is thereby considered to be essential for the bioactivity of these compounds. 

**Figure 4:**
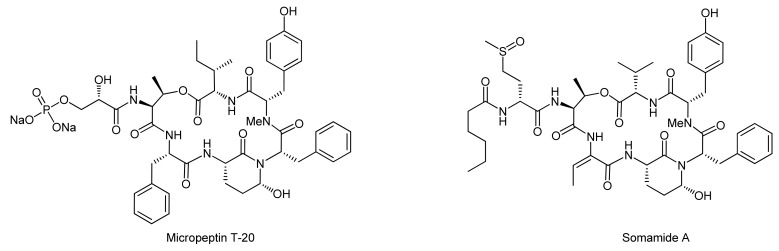
Micropeptin T-20 [63] and somamide A [64].

The first total syntheses of Ahp cyclodepsipeptides were undertaken by Shioiri and coworkers and succeeded in the syntheses of the potent chymotrypsin inhibitor Micropeptin T-20 isolated from *Microcystis aeruginosa* as well as somamide A that was isolated from *Lyngbya majuscula* (Figure 4) [63,64,65,66,67]. 

For the synthesis of the Ahp cyclodepsipeptide micropeptin T-20, Yokokawa and coworkers aimed at the establishment of a convergent, fragment-based synthesis approach [65]. To this end, the chemically instable Ahp unit was envisaged to be incorporated into the final molecules as a pentahomoserine derivate that upon oxidation of the alcohol and subsequent cyclization would lead to the Ahp hemiaminal moiety. However, the formation of this key unit was postponed to a late stage of the synthesis owing to the postulated sensitivity of the hemiaminals to the different reaction conditions. This perception was confirmed by extensive model reactions that proved that all attempts to construct the Ahp unit at fragment stages or in non-cyclized depsipeptides failed to provide an Ahp moiety [66]. 

In their retrosynthesis, Shioiri *et al*. therefore detached the glyceric acid phosphate from the threonine amine and fragmented the depsipeptide core into a tridepsipeptide moiety (Ile-O-Thr-Phe) and a tripeptide fragment consisting of pentahomoserine, phenylalanine and *N*-methyl tyrosine residue [65,66]. As the cyclization site, they chose the phenylalanine-pentahomoserine amide bond, firstly because of the higher reactivity of an amide compared to that of a hydroxyl group in a concurrent lactonization reaction; secondly, they assumed that the *cis*-amide bond formed by the *N*-methyltyrosine situated in the middle of a linear precursor would pre-arrange the molecule for an efficient cyclization reaction. 

**Scheme 18:**
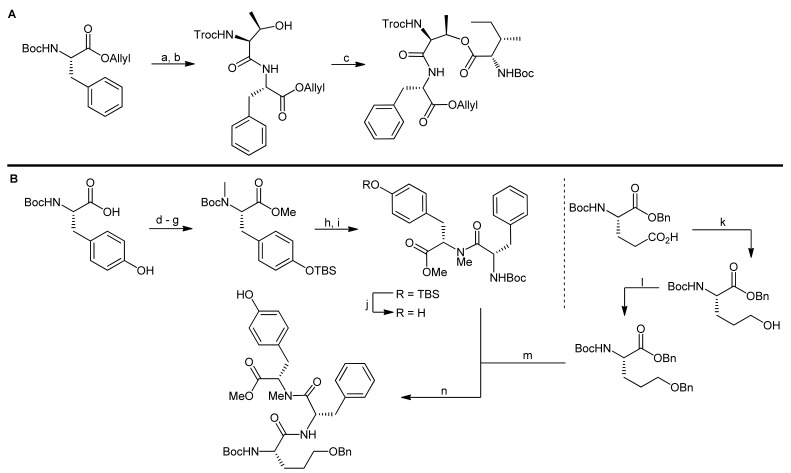
Synthesis of the micropeptin T-20 fragments [65,66].

The synthesis of the depsipeptide fragment was carried out in a straightforward manner starting from Boc-L-Phe-OAllyl (obtained by esterification of Boc-L-Phe-OH with allyl bromide) that was deprotected at the N-terminus using acidic conditions and then coupled to Troc-L-Thr-OH (obtained from the reaction of H-Ile-OH with TrocCl) using diethyl phosphorocyanidate (DEPC) as the activator (Scheme 18, **A**). The dipeptide was thereby obtained in a quantitative yield. Subsequently, the threonine side chain hydroxyl group was esterified with Boc-L-Ile-OH using EDC and DMAP as the reagents providing the desired depsipeptide fragment in 80% yield. 

The synthesis of the tripeptide fragment required the individual synthesis of the tyrosine and the pentahomoserine building blocks (Scheme 18, **B**). The synthesis of the *N*-methylated tyrosine started from Boc-L-Tyr-OH that was first protected at the phenol functionality with a TBS ether, then the *N*-methylation was carried out using *t*-butyl lithium and methyl iodide. Finally, the carboxyl group was protected as a methyl ester and the Boc group was removed for the coupling of the building block with Boc-L-Phe-OH. The coupling was carried out using BOP-Cl as the coupling reagent and afforded the dipeptide in 72% yield. 

**Scheme 19:**
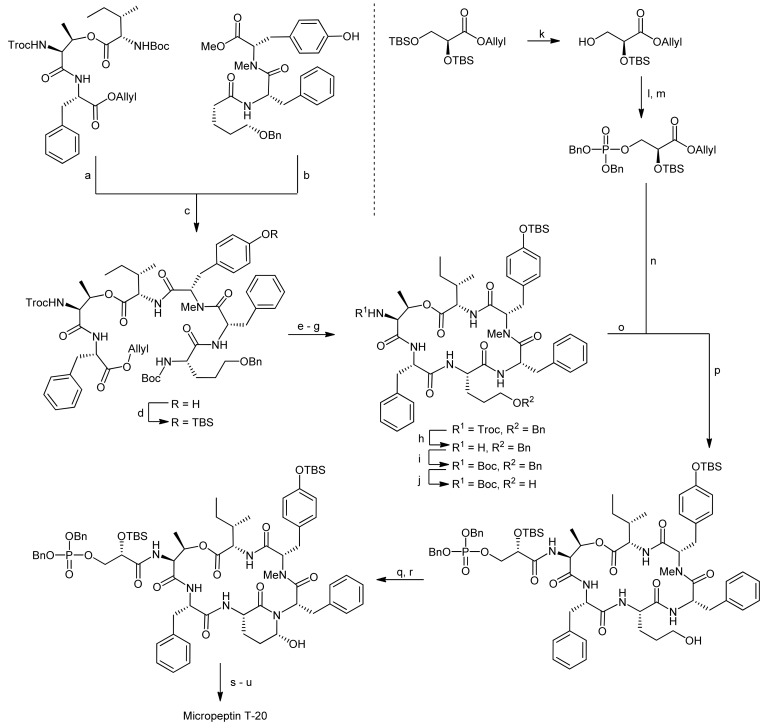
Assembly of the micropeptin T-20 framework and construction of the Ahp moiety [65,66].

The pentahomoserine building block required for the construction of the Ahp moiety was synthesized starting from Boc-L-glutamic acid benzyl ester. In the first step, the glutamic acid side chain was reduced using a two-step protocol to afford the alcohol in 76% yield. The hydroxyl function was then protected as a benzyl ether, thus yielding a fully protected pentahomoserine building block. After basic hydrolysis of the benzyl ester, the pentahomoserine residue was coupled to the TBS-deprotected Phe-NMe-Tyr dipeptide and the desired tripeptide was obtained after DEPC activation in a yield of 60%. After the deprotection of the individual fragments, the fused hexapeptide fragment was next obtained in a coupling reaction using again DEPC as the reagent (Scheme 19). To this end, first the TBS protection on the tyrosine was re-introduced. Sequential removal of the C-terminal allyl ester and the N-terminal Boc-group then set the stage for the macrocyclization reaction. Of all explored conditions, the best conversion was achieved with pentafluorophenyl diphenylphosphinate (FDPP) as the activating reagent in dichloromethane at high dilution conditions (2 mM). These conditions delivered the macrocycle in 84% yield. For completion of the synthesis, the permanent Troc protection on the threonine amine was removed and replaced by a Boc group; then, the benzyl ether protection of the pentahomoserine hydroxyl group was removed by hydrogenation. The Boc group was removed again and the resulting free amine was coupled with TBS-protected glyceryl phosphate benzyl ester. Having successfully installed the full cyclodepsipeptide framework, suitable methods for forming the Ahp moiety were next sought [65,66]. Dess-Martin oxidation provided the desired aldehyde in 50% yield; however, best yields were obtained with IBX oxidation that afforded the aldehyde in 80% yield. Subsequent treatment of the aldehyde with *p*-TsOH or PPTS caused decomposition of the intermediate, while stirring of the aldehyde in pH 6 phosphate buffer delivered an inseparable 1:1 mixture of the aldehyde and the desired hemiaminal. Treatment of this mixture with TBAF to remove the TBS protecting group however led to exclusive formation of the hemiaminal. Therefore, construction of the Ahp heminaminal was finally achieved by carrying out an IBX oxidation and then by treating the intermediate aldehyde with TBAF, resulting in the desired hemiaminal in 34% yield. To finally obtain micropeptin T-20, the benzyl ester protection of the phosphate was removed and the resulting material was first treated with pH 7 phosphate buffer to introduce sodium ions to the phosphate, followed by an ODS column purification to yield pure micropeptin T-20. The strategy developed for the synthesis of micropeptin T-20 was subsequently used to also synthesize the Ahp cyclodepsipeptide somamide A [67]. 

The Kaiser group subsequently set out to establish a solid-phase synthesis of Ahp cyclodepsipeptides [68,69]. As the previous solution syntheses of Ahp depsipeptides had shown that the Ahp unit forms spontaneously from a precursor glutamic aldehyde within the correctly assembled cyclodepsipeptide, a synthetic plan involving the solid-phase synthesis of the peptide backbone followed by selective formation of the glutamic aldehyde was developed. To prove this synthetic strategy, a synthesis of the chymotrypsin Ahp cyclodepsipeptide inhibitor symplocamide A was undertaken [70].

The key feature for their strategy was therefore the choice of an appropriate Ahp precursor that met the multiple requirements for their synthetic plan [68,69]. The precursor should (a) be compatible with Boc- and Fmoc-strategies, (b) generate the glutamic aldehyde upon cleavage from the resin and thereby form the Ahp hemiaminal and (c) carry a suitable anchoring group for the attachment to the solid-support to enable modifications on the C- and the N-terminus. To this end, they developed a new amino acid that incorporated a double bond as a masked glutamic aldehyde equivalent, enabling the oxidative cleavage to a glutamic aldehyde as reported by the Meldal group and a free acid as an anchoring point to the solid-support [71,72,73].

**Scheme 20:**
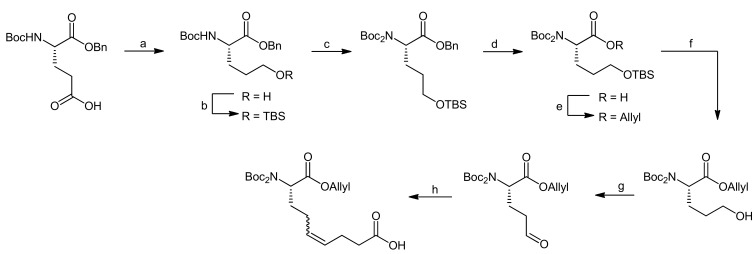
Synthesis of a general Ahp precursor molecule by the Kaiser group [68,69].

The synthesis of the Ahp precursor proceeded in 8 steps starting from Boc-L-glutamic acid benzyl ester (Scheme 20) [68]. NaBH_4_ reduction of the activated glutamic acid side chain was followed by protection of the resulting alcohol as a TBDMS ether. Then, a second Boc group was introduced to this intermediate in order to block the amine completely. After this transformation, the benzyl ester was removed by hydrogenation and replaced with an allyl protecting group. The subsequent removal of the TBDMS ether was performed under very mild copper-mediated conditions to prevent cleavage of the essential second Boc group. Dess-Martin oxidation afforded the aldehyde that could be converted to the final Ahp precursor molecule using a Wittig methodology.

**Scheme 21:**
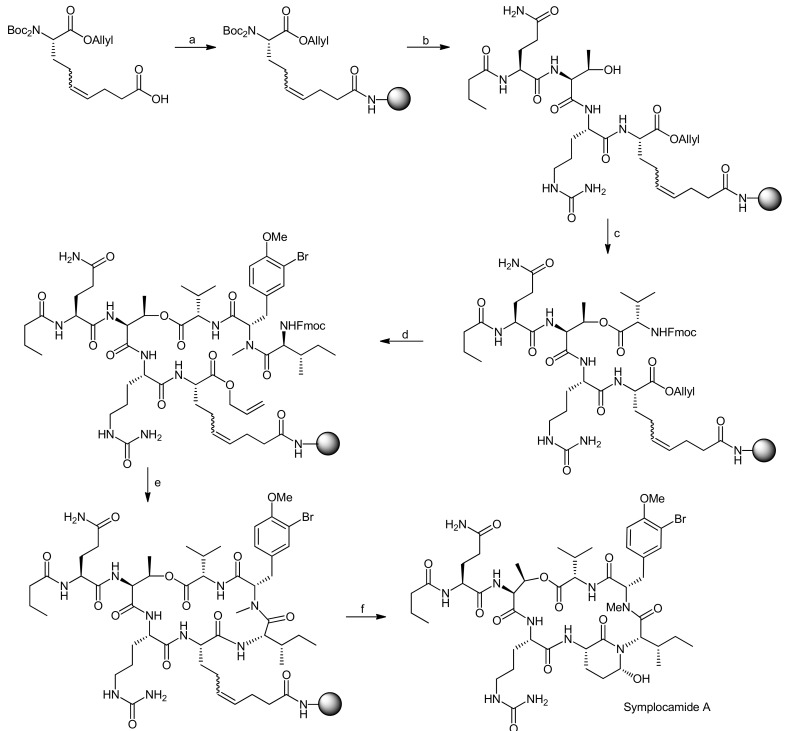
Solid-phase synthesis of symplocamide A [68,69].

After the successful generation of the Ahp precursor amino acid, the solid-phase synthesis was initiated (Scheme 21) [68,69]. The first step in this synthesis was the loading of the Ahp precursor onto the resin. In this case, the utilization of a PEG-based resin (NovaPEG resin) became necessary because regular, polystyrene-based resins had not been stable to the cleavage conditions. To create optimal conditions for the on-resin macrolactamization reaction and to prevent side reactions during assembly of the cylodepsipeptide, the resin was only partially loaded.

After a capping step, the Boc groups were cleaved and the next amino acid was coupled. In general, amino acids were coupled via HBTU/HOBt activation and the N-terminal Fmoc or Boc protecting groups were cleaved with piperidine or TFA, respectively. The first coupled amino acid was Fmoc-citrulline-OH, followed by Fmoc-Thr-OH, Boc-Glu-OH and finally butyric acid to yield the esterification precursor. The esterification of the threonine side chain with Fmoc-Val-OH was achieved with DCC/DMAP activation in 4 × two hours, choosing these shorter and multiple reactions in favor of one long reaction to prevent racemization and to optimize the coupling rate. The resulting depsipeptide was then deprotected and coupled with a *N*-Boc-*N,O*-dimethyl-3-bromotyrosine building block using HBTU/HOBt activation. The removal of the Boc group from the N-terminus then yielded an N-methyl intermediate to which Fmoc-Ile-OH could be coupled using PyBrOP activation. Fmoc and allyl deprotection using Pd(PPh_3_)_4_/morpholine yielded the macrolactamization precursor. The macrocycle was obtained by using PyBOP/HOBt activation and an elongated reaction time. 

The conversion of the double bond to the aldehyde and simultaneous cleavage from the resin was achieved using OsO_4_ and NaIO_4_ while DABCO was added to suppress hydroxymethyl ketone formation (Scheme 21). The described conditions thereby represent a new safety-catch cleavage method for solid-phase synthesis. Symplocamide A was obtained with an overall yield of 3% (over all steps); interestingly, the newly developed solid-phase approach turned out as robust and flexible enough to also allow the synthesis of other custom-made Ahp cyclodepsipeptides. 

## 6. Conclusions

The case studies compiled in this review illustrate the challenges that have to be met when attempting the synthesis of a cyclodepsipeptide natural product. Despite the vast number of available methods for the establishment of peptide and ester bonds, their syntheses are far from routine work as many hurdles such as stereocontrol, handling of delicate moieties, formation of macrocyclic structures or adaption of reaction conditions to a solid-phase synthesis are far from being solved on a general level and clearly further research efforts into this direction are still required. 

Most syntheses of cyclodepsipeptide natural products remain unique solutions, adapted to the structure of interest. In this review we have illustrated some approaches to more general routes to the syntheses of cyclodepsipeptide natural products and analogs thereof that will hopefully be an inspiration to drive forward the research in this field. Given the potent bioactivities found in cyclodepsipeptide natural products, we are optimistic that more general approaches will flourish driven by the desire to implement cyclodepsipeptide natural products as lead structures in medicinal chemistry research.

## Figures and Tables

*Reagents and conditions*: (a) Et_3_N, CH_2_Cl_2_, reflux, acrylic acid (71%); (b) Grubbs’ catalyst, CH_2_Cl_2_ (97%); (c) Me_2_CuCNLi_2_, Et_2_O, −78 °C (86%); (d) LiAlH_4_, Et_2_O, 0 °C (83%); (e) TBSCl, imidazole, CH_2_Cl_2_ (98%); (f) Boc-Pro-OH, Yamaguchi esterification (91%); (g) TBAF, THF (88%); (h) TPAP, NMO, 4Å MS, CH_2_Cl_2_ (89%); (i) Me_2_NEt, *c*-Hex_2_BCl, Et_2_O, −78 °C to −20 °C; (j) TBSOTf, 2,6-lutidine, CH_2_Cl_2_, −50 °C (74%, 2 steps); (k) K_2_CO_3_, MeOH; (l) NaIO_4_, MeOH, buffer (75%, 2 steps).

*Reagents and conditions*: (a) TFA, CH_2_Cl_2_; (b) Boc-NMe-Ala-OH, PyAOP, DIEA, CH_2_Cl_2_ (79%, 2 steps); (c) Boc-Tyr(OMe)-OH, PyAOP, DIEA, CH_2_Cl_2_ (95%); (d) PyAOP, DIEA, CH_2_Cl_2_ (76%); (e) HF-pyr, THF (98%); (f) DIAD, Ph_3_P, AcSH (85%); (g) K_2_CO_3_, MeOH, 0 °C; (h) prolyl-polyketide fragment, DPPA, Et_3_N, CH_2_Cl_2_ (80%, 2 steps); (i) DDQ, CH_2_Cl_2_, H_2_O, 0 °C to RT (93%); (j) DPPA, PPh_3_, DIAD, THF (97%); (k) HF-pyr, THF; (l) TESOTf, 2,6-lutidine, CH_2_Cl_2_, −78 °C (86%, 2 steps); (m) Ph_3_P, THF, 50 °C (63%); (n) TBSOTf, 2,6-lutidine, CH_2_Cl_2_; (o) TBAF, THF 0 °C, (86%, 2 steps) (p) LiOH, *t*-BuOH, THF, H_2_O; (q) PyAOP, CH_2_Cl_2_, DIEA (73%, 2 steps); (r) HF-pyr, THF (65%).

*Reagents and conditions*: (a) MPMimidate, TfOH, THF, 0 °C (b) vinylmagnesium bromide, THF, 0 °C (87%, 2 steps); (c) PhSiOReO_3_, BSA, ether, 0 °C; (d) Et_3_N, K_2_CO_3_, MeOH (42% (*E*) and 44% (*Z*), 2 steps); (e) 2 mol% Ru(OAc)_2_[(*S*)-binap], H_2_ (100 atm), MeOH, 50 °C (quant.); (f) 4Å MS, toluene, −78 °C (95% (>95% ds)); (g) TrocCl, DMAP, pyridine, CH_2_Cl_2_, 0 °C, (quant.); (h) DDQ, H_2_O, CH_2_Cl_2_; (i) Fmoc-Pro-OH, C_6_H_2_Cl_3_COCl, DIEA, DMAP, toluene (92%, 2 steps); (j) OsO_4_, oxone, NaHCO_3_, DMF then NaIO_4_, H_2_O, *t*-BuOH (79%).

*Reagents and conditions*: (a) EDC, HOAt, DIEA, CH_2_Cl_2_, 0 °C to RT (81%); (b) Tf_2_O, Ph_3_PO, CH_2_Cl_2_, 0 °C; (c) Zn, NH_4_OAc, THF; (d) Pd(Ph_3_P)_4_, *N*-methylaniline, THF (86%, 3 steps); (e) Et_2_NH, CH_3_CN; (f) HATU, DIEA, CH_2_Cl_2_ (75%, 2 steps); (g) Pd(Ph_3_P)_4_, *N-*methylaniline, THF; (h) Et_2_NH, CH_3_CN; (i) HATU, DIEA, CH_2_Cl_2_ (1.0 mM) (72%, 3 steps).

*Reagents and conditions*: (a) BuLi, Cp_2_ZrCl_2_, THF, −78 °C to RT (55% (93% ds)); (b) basic hydrolysis (NaOH); (c) carbonyldiimidazole, (EtO_2_CCH_2_CO_2_)_2_Mg, THF; (d) NaBH_4_, THF, MeOH (84:16); (e) LiOH, THF, H_2_O; recrystallization (34%, 3 steps); (f) K_2_CO_3_, allyl bromide; (g) 4 M HCl in EtOAc; (h) Fmoc-d-Cys(S-Trt)-OH, EDC, HOBt, DIEA (76%, 3 steps); (i) Et_2_NH; (j) Fmoc-d-Ala-OH, EDC, HOBt, DIEA (79%, 2 steps); (k) (*S*)-3-hydroxy-7-mercaptohept-4-enoic acid, PyBOP, DIEA (65%); (l) Pd(PPh_3_)_4_, morpholine, MeOH (87%); (m) 2-methyl-6-nitrobenzoic anhydride, DMAP, CH_2_Cl_2_ (67%); (n) I_2_, CH_2_Cl_2_, MeOH (quant.).

*Reagents and conditions*: (a) TrtSH, Cs_2_CO_3_, THF, 0 °C to RT (78%); (b) DIBAL-H, CH_2_Cl_2_, −78 °C to −30 °C (91%); (c) (COCl)_2_, DMSO, Et_3_N, CH_2_Cl_2_, −78 °C to −10 °C (74%); (d) Ti(IV) catalyst, toluene, 4 °C; (e) TBAF, THF (95% (>98%ee), 2 steps); (f) LiOH, MeOH (quant.).

*Reagents and conditions*: (a) Tos_2_O, pyridine, 0 °C (97%); (b) DABCO, CH_3_CN then Et_2_NH (96%); (c) BOP, DIEA, CH_3_CN, CH_2_Cl_2_ (98%); (d) LiOH, THF, H_2_O (93%); (e) DIAD, PPh_3_, TsOH, THF, 0 °C (62%); (f) I_2_, MeOH (84%).

*Reagents and conditions*: (a) *i*-PrMgCl, HN(OMe)Me, THF, 0 °C (67%); (b) BuLi, THF, −78 to 0 °C, (75%); (c) (*R*,*R*)-catalyst (10 mol%), *i*-PrOH; (d) Red-Al, ether, 0 °C to RT, (58%, 2 steps); (e) TBAF, THF; (f) TsCl, DMAP, CH_2_Cl_2_ (70%, 2 steps); (g) LiBF_4_, CH_3_CN-H_2_O; (h) NaClO_2_, NaH_2_PO_4_, 2-methyl-2-butene; (i) TrtSH, *t*-BuOK, THF, 0 °C (65%, 3 steps).

*Reagents and conditions*: (a) TrtSH, Et_3_N, CH_2_Cl_2_; (b) Ph_3_P=CH-CHO, benzene, reflux (60%, 2 steps); (c) TiCl_4_, DIEA, CH_2_Cl_2_; (d) LiOH, THF, H_2_O (76%, 2 steps); (e) TMSEOH, DCC, CH_2_Cl_2_ (80%).

*Reagents and conditions*: (a) H-Thr-OMe*HCl, PyBOP, DIEA, CH_2_Cl_2_ (97%); (b) piperidine, CH_2_Cl_2_; (c) Fmoc-d-Val-OH, PyBOP, DIEA, CH_2_Cl_2_ (82%, 2 steps); (d) MsCl, Et_3_N, CH_2_Cl_2_; (e) DABCO, piperidine, CH_2_Cl_2_ (74%, 2 steps); (f) LiOH, THF, H_2_O; (g) Fmoc-Cl, THF, H_2_O (64%, 2 steps); (h) mercaptoheptenoic acid building block (Scheme 12), DCC, CH_2_Cl_2_ (85%); (i) tripeptide (from **A**), PyBOP, DIEA, CH_2_Cl_2_ (75%); (j) TBAF, THF; (k) HATU, DIEA, CH_2_Cl_2_ (54%, 3 steps); (l) I_2_, MeOH, CH_2_Cl_2_ (50%).

*Reagents and conditions*: (a) HOAt, EDC, NaHCO_3_, CH_2_Cl_2_, DMF, 0 °C; (b) 10% Pd/C, H_2_, MeOH; (c) 4 N HCl, EtOAc; (d) DEPBT, NaHCO_3_, THF, 0 °C (83%, 2 steps); (e) HOAt, EDC, DMF (76%); (f) 4 N HCl, dioxane.

*Reagents and conditions*: (a) benzyl carbamate, *t*-BuOCl, NaOH (aq.), (DHQD)_2_PHAL, K_2_OsO_2_(OH)_4_, *n*-PrOH, 0 °C (71%, (99% ee)); (b) TBSOTf, 2,6-lutidine, CH_2_Cl_2_, 0 °C (98%); (c) 10% Pd/C, Boc_2_O, H_2_, MeOH (97%); (d) NH_3_, MeOH (99%); (e) NaIO_4_ (aq.), 5% RuCl_3_, NaHCO_3_, EtOAc, CH_3_CN (65%); (f) BnBr, NaHCO_3_, DMF (84%); (g) i. 4N HCl, EtOAc; ii. FmocCl, NaHCO_3_, dioxane, H_2_O (92%); (h) TrtOH, cat. H_2_SO_4_, Ac_2_O, HOAc (71%); (i) piperidine, CH_2_Cl_2_ (quant.); (j) Fmoc-L-Asn(Trt)-OH, HOAt, EDC, NaHCO_3_, CH_2_Cl_2_, DMF, 0 °C (90%); (k) Boc-L-Chp(TBS)-OH, EDC, DMAP, CH_2_Cl_2_, 0 °C (87%); (l) *B*-bromocatecholborane, CH_2_Cl_2_, 0 °C (m) Boc-L-Leu-D-Ala-OH, HOAt, EDC, CH_2_Cl_2_, DMF, 0 °C, (81%, 2 steps); (n) TBAF, HOAc, EtOAc (95%); (o) 10% Pd/C, H_2_, EtOH (94%).

*Reagents and conditions*: (a) DEPBT, NaHCO_3_, THF, 0 °C (82%); (b) 4 N HCl, EtOAc; (c) Boc-D-Orn(SES)OH, HOAt, EDC, NaHCO_3_, CH_2_Cl_2_, DMF, 0 °C to 15 °C (90%, 2 steps); (d) 10% Pd/C, H_2_, MeOH (98%); (e) H-L-Hpg-Gly-OBn, HOAt, EDC, NaHCO_3_, CH_2_Cl_2_, DMF, 0 °C (90%); (f) 10% Pd/C, H_2_, MeOH (90%).

*Reagents and conditions*: (a) DEPBT, NaHCO_3_, DMF, 0 °C (50%–68%); (b) i. *B*-bromo-catecholborane, CH_2_Cl_2_; ii. heptapeptide, HOAt, EDC, DMF, 0 °C (60%–82%); (c) i. *B*-bromocatecholborane, CH_3_CN; ii. 10% Pd/C, H_2_, EtOH; iii. HOAt, EDC, CH_2_Cl_2_, DMF, 0 °C (54%–72%); (d) Bu_4_NF, *i*-PrOH, DMF; (e) (2*Z*,4*E*)-7-methyl-2,4-octadienoic anhydride, CH_2_Cl_2_, DMF (69%, 2 steps); (f) HF, anisole, −78 °C to 0 °C (82%); (g) H_2_O, TFA (18%); (h) HF, anisole, −78 °C to 0 °C (83%).

*Reagents and conditions*: (a) 4 N HCl, EtOAc; (b) Troc-L-Thr-OH, DEPC, Et_3_N, DMF (quant., 2 steps); (c) Boc-L-Ile-OH, EDC, DMAP, THF (80%); (d) TBSCl, imidazole, DMF; (e) K_2_CO_3_, MeOH, H_2_O (86%, 2 steps); (f) *t*-butyl lithium, MeI, THF (84%); (g) MeI, KHCO_3_, DMF (94%); (h) 4 N HCl, EtOAc; (i) Boc-l-Phe-OH, BOPCl, Et_3_N, CH_2_Cl_2_ (72%, 2 steps); (j) TBAF, THF (quant.); (k) i. EtOCOCl, Et_3_N, THF; ii. NaBH_4_, H_2_O (76%); (l) BnBr, Ag_2_O, Et_2_O (82%); (m) 0.5 N aq. LiOH, THF, (n) DEPC, Et_3_N, DMF (60%, 3 steps).

*Reagents and conditions*: (a) 4 N HCl, dioxane; (b) 0.5 N aq. LiOH, THF; (c) DEPC, Et_3_N, DMF (77%, 3 steps); (d) TBSCl, imidazole, DMF (75%); (e) Pd(PPh_3_)_4_, morpholine, THF; (f) 4 N HCl, dioxane (g) FDPP, DIEA, CH_2_Cl_2_ (84%, 3 steps); (h) Zn, AcOH, THF; (i) Boc_2_O, Et_3_N, dioxane, H_2_O (77%, 2 steps); (j) H_2_, 20% Pd(OH)_2_, EtOAc (quant.); (k) CeCl_3_*7H_2_O, NaI, CH_3_CN (62%); (l) (BnO)_2_P-*N*-*i*-Pr_2_, tetrazole, CH_2_Cl_2_; (m) *m*-CPBA (quant., 2 steps) (n) Pd(PPh_3_)_4_, morpholine, THF; (o) 4 N HCl, dioxane, (p) DEPC, Et_3_N, DMF (57%, 3 steps); (q) IBX, DMSO; (r) TBAF, THF (34%, 2 steps); (s) H_2_ 10% Pd-C, 90% aq. EtOH; (t) pH 7 phosphate buffer-MeOH; (u) purification on ODS column (77%, 3 steps).

*Reagents and conditions*: (a) i. EtOCOCl, Et_3_N, THF, −15 °C, ii. NaBH_4_, THF, H_2_O, −15 °C to RT (84%); (b) imidazole, TBSCl, CH_2_Cl_2_, 0 °C to RT (95%); (c) Boc_2_O, DMAP, CH_3_CN (81% b.r.s.m.); (d) 10% Pd/C, H_2_, EtOAc (99%); (e) i. Cs_2_CO_3_, MeOH; ii. allylbromide, DMF (95%); (f) CuCl_2_*H_2_O, acetone, H_2_O, reflux (92%); (g) Dess-Martin periodinane, CH_2_Cl_2_, (84%); (h) Ph_3_P(CH_2_)_3_COOH, LHMDS, THF, 0 °C to RT (64%).

*Reagents and conditions*: (a) i. amino NovaPEG resin, HOBt, HBTU, DIEA, CH_2_Cl_2_, DMF; ii. CH_2_Cl_2_, DIEA, Ac_2_O (3:1:1); (b) i. TFA, CH_2_Cl_2_ (1:1); ii. Et_3_N, CH_2_Cl_2_ (1:9); iii. Fmoc-Cit-OH, HOBt, HBTU, DIEA, DMF (30%, by Fmoc determination, two steps); iv. piperidine, DMF (1:4); v. Fmoc-Thr-OH, HOBT, HBTU, DIEA, DMF; vi. piperidine, DMF (1:4); vii. Fmoc-Gln(Trt)-OH, HOBT, HBTU, DIEA, DMF (88% by Fmoc determination, two couplings); viii. piperidine, DMF (1:4); ix. butyric acid, HOBT, HBTU, DIEA, DMF; (c) Fmoc-Val-OH, DCC, DMAP, CH_2_Cl_2_, DMF (79% by Fmoc determination, two couplings); (d) i. piperidine, DMF (1:4); ii. *N*-Boc-*N*,*O*-dimethyl-3-bromotyrosine, HOBT, HBTU, DIEA, DMF; iii. TFA, CH_2_Cl_2_ (1:1); iv. Et_3_N, CH_2_Cl_2_ (1:9); v. Fmoc-Ile-OH, PyBrOP, DIEA, DMF (60%, by Fmoc determination, two couplings); (e) i. piperidine, DMF; ii. Pd(PPh_3_)_4_, morpholine, CH_2_Cl_2_; iii. PyBOP, HOBt, DIEA, DMF; (f) OsO_4_, NaIO_4_, DABCO, *t*-BuOH, THF, H_2_O (3% overall yield for whole solid phase synthesis).
